# Morphological and Molecular Characterizations of *Musa* (ABB) ‘Mali-Ong’ in Thailand

**DOI:** 10.3390/biology11101429

**Published:** 2022-09-29

**Authors:** Duangporn Premjet, Thanita Boonsrangsom, Kawee Sujipuli, Kumrop Rattanasut, Anupan Kongbungkerd, Siripong Premjet

**Affiliations:** 1 Center of Excellence in Research for Agricultural Biotechnology, Faculty of Agriculture, Natural Resources and Environment, Naresuan University, Muang, Phitsanulok 65000, Thailand; 2 Department of Agricultural Science, Faculty of Agriculture, Natural Resources and Environment, Naresuan University, Muang, Phitsanulok 65000, Thailand; 3 Department of Biology, Faculty of Science, Naresuan University, Muang, Phitsanulok 65000, Thailand

**Keywords:** banana, morphological traits, genetic variation, inter simple sequence repeat, ‘Kluai Nam Wa’, sequence-related amplified polymorphism

## Abstract

**Simple Summary:**

The banana cultivar *Musa* (ABB) ‘Mali-Ong’ is widely used in the processed food industry in Thailand, where it determines the quality of the products. However, the sub-cultivars of ‘Nam Wa Mali-Ong’ are almost indistinguishable, with few morphological differences and minimal genetic variation. This study used 77 morphological characteristics and two types of molecular markers to distinguish Nam Wa Mali-Ong from other cultivars. The study also assessed the genetic variation of nine ‘Nam Wa Mali-Ong’ clones and compared them with 10 other samples of bananas with different genomes or chromosome sets. The molecular markers grouped the ‘Nam Wa Mali-Ong’ samples. Four clones (A, B, D, and I) were superior and had higher bunch weights. This study will be useful for germplasm evaluation and future ‘Nam Wa Mali-Ong’ improvements.

**Abstract:**

*Musa* (ABB) ‘Mali-Ong’ is an economically important banana cultivar in Thailand. We morphologically and molecularly characterized ‘Nam Wa Mali-Ong’. Leaf blade width was the only statistically different morphological character among the clones. To determine genetic variation, nine ‘Nam Wa Mali-Ong’ clones were compared with 10 samples of *Musa* ABB, AA, and BB cultivars by fingerprinting using seven pairs of sequence-related amplified polymorphism (SRAP) and eight inter simple sequence repeat (ISSR) markers. The SRAP and ISSR primers generated 65 and 76 amplicons, respectively, of which 57 (87.7%) and 62 (81.6%) amplicons, respectively, were polymorphic; the polymorphic information content was 0.28–0.49. The SRAP data revealed two distinct groups: Group I, comprising two subgroups (one including all ABB samples and the other containing the BB genome accessions), and Group II, comprising the AA genome accessions. The ISSR data revealed two groups: Group I, which incorporated the AA (Hom Champa) genome, and Group II, consisting of two subgroups: Subgroup A, comprising only the AA (Hom Chan) accessions, and subgroup B, comprising all the ABB accessions and wild banana *M. balbisiana* (BB genome). The ‘Nam Wa Mali-Ong’ samples clustered together, regardless of the markers used. SRAP and ISSR markers will be useful for germplasm evaluation and future *Musa* (ABB) improvements.

## 1. Introduction

Banana (*Musa* sp.) is an economically important fruit grown in several tropical countries with a high demand in global markets. In 2021, approximately 100 million tons of bananas were supplied from across over 130 tropical nations [1]. Banana has a high nutritional value and numerous health benefits. It is rich in dietary fiber, potassium, antioxidants, vitamins B6 and C, and phytonutrients. Bananas are widely consumed fresh and processed to produce sun-dried bananas, banana flour, banana starch, and various snacks [2]. Edible banana cultivars are almost exclusively the result of interspecific crosses between two wild species. A cross between *Musa acuminata* (AA genome) and *Musa balbisiana* (BB genome) produces the AAB, ABB, AABB, and ABBB genome groups. The triploids (AAA and AAB cultivars) are cultivated globally and play a significant role in the global economy [3]. The ABB cultivar originated in South-East Asia and India and is divided into nine subgroups: Bluggo, Monthan, Ney Mannan, Pisang Awak, Peyan, Pelipita, Saba, Kalapua, and Kloe Teparod [4]. In Thailand, banana (especially the ABB cultivar) is the most important fruit crop because of its high biotic and abiotic stress resistance. Moreover, bananas contain polyphenols that can act as health-promoting substances and exhibit an anti-diabetic effect [5]. Many banana cultivars with ABB genomes, such as ‘Kluai Tip Yai’, ‘Kluai Hak Muk’, and ‘Kluai Nam Wa,’ are grown in Thailand. The ‘Kluai Nam Wa’ cultivar is extremely valuable to the banana processing business [6]. It is widely planted in northern Thailand, especially in the provinces of Phitsanulok and Nan. Kluai Nam Wa is sweet and can be eaten fresh without heat treatment [7]. The fruits are nutritious and processed as high-quality sundried bananas, with a yellowish-brown color, sweet flavor, and soft texture. Many sub-cultivars, such as Kluai Nam Wa Deang, ‘Kluai Nam Wa Khwan’, and ‘Kluai Nam Wa Mali-Ong’, belong to the *Musa* (ABB genomes) complex. These sub-cultivars are almost indistinguishable, with few morphological differences and minimal genetic variation. Therefore, identifying the ‘Kluai Nam Wa’ sub-cultivars remains difficult. To date, scarce scientific information is available on the conservation and utilization of ‘Kluai Nam Wa Mali-Ong’ and its morphology and phylogenetic relationship. The definition of genetic variation or cultivar is the foundational step in germplasm management, not only for identifying features but also for facilitating their suitable application [8]. Characterization is suggested using morphotaxonomy, cytology, and molecular genotyping, including of nutritional traits [9]. Molecular markers, including random amplified polymorphic DNA (RAPD), simple sequence repeat (SSR), simple sequence repeat (ISSR), and sequence-related amplified polymorphism (SRAP) markers, have been employed to examine genetic diversity among banana accessions and species [2,10,11,12]. SRAP markers have been used to determine the relationship of a cross between *Musa* (ABB) and two wild cultivars (AA and BB) [13]. However, no precise information exists on the morphological traits of ‘Kluai Nam Wa Mali-Ong’. This cultivar is the most important banana for the food industry in Thailand and determines the quality of the products. The collection of clones distributed throughout Phitsanulok in northern Thailand is essential for establishing ex situ conservation of ‘Kluai Nam Wa Mali-Ong’ to preserve an elite cultivar that will strengthen and sustainably drive the bio, circular, and green economy. The objectives of this study were: (1) to collect nine ‘Kluai Nam Wa Mali-Ong’ clones from a farm in Phitsanulok to describe their morphological characteristics; and (2) to evaluate the genetic variation of ‘Kluai Nam Wa Mali-Ong’ collected from agricultural fields in nine districts of Phitsanulok Province in Thailand using SRAP and ISSR markers.

## 2. Materials and Methods

### 2.1. Collection of Plant Materials

**Figure 1:**
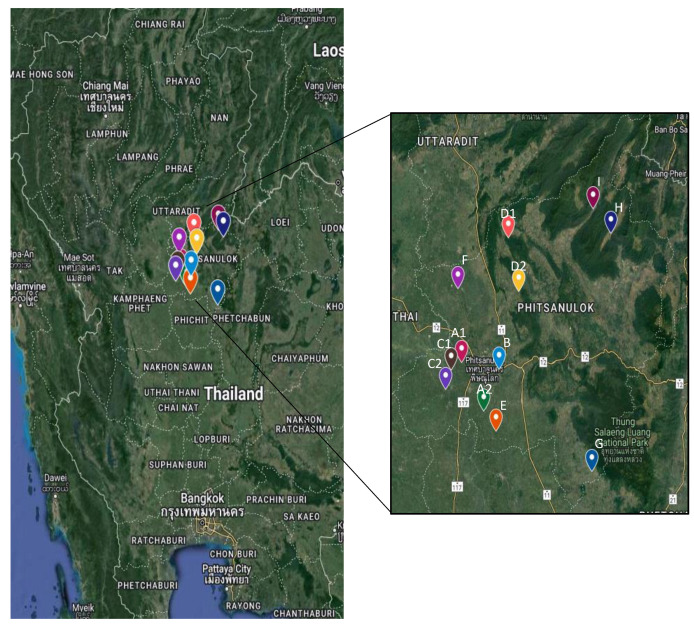
Twelve collection sites of *Musa* (ABB) ‘Mali-Ong’ across nine districts of Phitsanulok Province, Thailand. A: Mueang; B: Wang Thong; C: Bang Rakam; D: Wat Bot; E: Bang Krathum; F: Phrom Phiram; G: Noen Maprange; H: Nakhon Thai; and I: Chat Trakan.

Ten sword suckers of *Musa* (ABB) ‘Mali-Ong’ were collected in February 2021 from a farm in each of the 12 plantation areas in nine districts of Phitsanulok Province: Muang, Bang Rakam, Bang Kratum, Nakhon Thai, Chat Trakarn, Wang Thong, Neon-Maprang, Wat Bot, and Prom Phiram (Figure 1). *Musa* reference genomes (AA, BB, and ABB) were obtained from the banana germplasm at the Nakhon Ratchasima and Sukhothai Horticultural Research Center. Banana samples were coded as shown in Table 1.

### 2.2. Observation of Agronomic Traits

‘Kluai Nam Wa Mali-Ong’ accessions were cultivated with a 4 × 4 m spacing in a field at Plant Propagation Center No. 6, Phitsanulok, Thailand. The banana plants were provided with 17–20 L of water per day using a drip water system. Compost (1 kg plant^−1^) was applied once a month for 7 months, and chemical fertilizer (15-15-15) was applied 7 months after transplantation at a rate of 125 g plant^−1^. The bunch weight (kg) and the number of hands/bunch were recorded.

### 2.3. Voucher Deposition of Musa (ABB) ‘Mali-Ong’

The herbarium in the Department of Biology, Naresuan University, confirmed the botanical identity of the plants. Samples of *Musa* (ABB) ‘Mali-Ong’ were deposited at the Naresuan University and Laboratory Herbarium (herbarium deposit vouchers 05780 and 05781) for future reference. 

### 2.4. Determination of Morphological Characteristics

We determined the morphological parameters of *Musa* (ABB) ‘Mali-Ong’ cultivated under standardized conditions from 8–16 months (plant to fruit). A morphological record was made according to the 1996 Book of Descriptors for Banana (*Musa* spp.) [14]. The following 77 characteristics were recorded: 7 features of the artificial trunk (pseudostem), 17 of the leaf, 9 of the inflorescence/plant (male bud), 8 of the bracts, 16 of the male flowers, and 20 of the fruit. The Royal Horticultural Society Color Charts were used to specify colors. Three samples of each clone were evaluated for their qualitative and quantitative characteristics. The ‘Nam Wa Mali-Ong’clones were named as follows: clone A, Muang; clone B, Wang Thong; clone C, Bang Rakam; clone D, Wat Bot; clone E, Bang Kratum; clone F, Prom Phiram; clone G, Neon-Maprang; clone H, Nakhon Thai; and clone I, Chat Trakarn. 

### 2.5. Genetic Variation Assessment Using Molecular Markers

#### 2.5.1. Primers

Eight ISSR primers and seven SRAP marker pairs (Table 2 and Table 3) were purchased from Gibthai Co., Ltd. (Bangkok, Thailand). 

#### 2.5.2. Genomic DNA Extraction

Six months after planting, the cigar leaves of the sample plants were used for DNA extraction. Two samples were obtained from each collection site for DNA extraction. Genomic DNA was extracted using the PureDireX Genomic DNA Isolation Kit (Plant) (Bio-Helix Co., Ltd., New Taipei City, Taiwan) according to the manufacturer’s instructions. Briefly, 100 mg of banana leaves were pulverized in liquid nitrogen to a powdered form, and 500 µL of buffer PL and 2.5 µL of RNase A (10 mg mL^−1^) were added to the powder in 1.5 mL tubes and stirred gently. The tubes were incubated for 60 min at 75 °C and centrifuged at 14,462× *g* for 5 min. The clear top layer was transferred to a new 1.5 mL tube, and an equal amount of cold isopropanol was added. Subsequently, 400 µL of buffer W1 and the mixture were added to a column PC and centrifuged at 14,462× *g* for 30 s. The column PC was removed from its container and returned to the original collection tube after centrifugation. The centrifuged flowthrough was discarded. To retrieve DNA, 600 µL of W2 buffer (ethanol:buffer = 1:4) was poured into column PC and centrifuged for 2 min at 14,462× *g*. The DNA precipitate was preserved at −20 °C until further use.

#### 2.5.3. DNA Quality and Quantity Determination

The quantity and purity of the DNA were measured using 0.8% agarose gel in a 1× TAE buffer and RedSafeTM nucleic acid staining solution. The intensity of the ultraviolet fluorescent bands was proportional to the amount of DNA. The intensity was compared with a known standard DNA ladder of 100 bp (ONEMARK 100 DNA bp ladder, Bio-Helix Co. Ltd., New Taipei City, Taiwan) [13].

#### 2.5.4. ISSR-PCR

Twenty ISSR primers were tested, but only eight ISSR primers provided clear bands. These eight ISSR primers were used for polymerase chain reaction (PCR) amplification [11,15,16,17,18,19]. The 20 µL reaction mixture consisted of 10× PCR buffer, MgCl_2_ (0.2 mM), Taq polymerase (1.25 U), and 50 ng genomic DNA. A thermal cycler was used to perform DNA amplification (T100^TM^, Bio-Rad, Hercules, CA, USA). An initial denaturation at 94 °C for 5 min was followed by 35 cycles of denaturation for 30 s at 94 °C, annealing for 1 min at 50–53 °C, extension for 1 min at 72 °C, and a final extension for 2 min at 72 °C [13].

#### 2.5.5. SRAP-PCR

Sixty-four pairs (eight forward primers: Me1, Me2, Me3, Me4, Me5, Me6, Me7, and Me9; and eight reverse primers: Em1, Em2, Em3, Em4, Em5, Em6, Em7, and Em8) were screened to generate clear DNA bands. A total of 20 µL of 2× OnePCR^TM^ Plus (Bio-Helix Co., Ltd., New Taipei City, Taiwan), including Taq DNA polymerase, dNTP mix and gel loading dye, 5 M SRAP primers, and 50 ng of genomic DNA, was used for the PCR experiments. Thermal cycler amplifications were carried out using a denaturation step at 94 °C for 3 min, followed by 5 cycles of 3 steps: 1 min for denaturation at 94 °C, 1 min for annealing at 35 °C, and 2 min for an extension at 72 °C, followed by 35 cycles with an annealing temperature of 50 °C and a final prolongation step for 5 min at 72 °C [13].

The ISSR and SRAP-PCR amplicons were separated on 1.5% (*w*/*v*) agarose gel plates, stained with RedSafe^TM^, intron biotechnology, Inc., Gyeonggi, Korea and visualized using a gel documentation system (Thermo Fisher Scientific, Taoyuan City, Taiwan). The size of each fragment was estimated using the ONEMARK 100 DNA Ladder (Bio-Helix Co., Ltd., New Taipei City, Taiwan). Gel documentation systems were used to observe the ISSR and SRAP-PCR amplicons on an agarose gel stained with RedSafe^TM^, intron biotechnology, Inc., Gyeonggi, Korea. This DNA Ladder (ONEMARK 100) was used to assess the size of each of the fragments (Bio-Helix Co., Ltd., New Taipei City, Taiwan). 

### 2.6. Analysis of Morphological Data

The mean and standard deviation were presented for the quantitative morphological parameters. Analysis of variance (ANOVA) was performed to examine whether the results from the nine clones were significant using a randomized plot design and one-way ANOVA. The DMRT test (*p* < 0.05) investigated significant differences between the mean values.

### 2.7. Molecular Data Analysis

To build a binary matrix, the SRAP and ISSR amplicons were scored as either 1 (present) or 0 (absent). Polymorphism (%) and the average number of bands per primer were computed from the total number of amplified bands. The polymorphic information content (PIC) was calculated using the following formula: PIC =$\;1-\sum_{Pi}2$, where *Pi* is the *i*th allele frequency for an individual population [20]. Free Tree & Tree View, a freeware tool, was used to build phylogenetic trees using the unweighted pair-group method with arithmetic average (UPGMA) analysis based on Dice similarity coefficients, and a bootstrap analysis based on 1000 bootstrap repeats was performed [21].

## 3. Results

### 3.1. Morphological Characteristics of Musa (ABB) ‘Mali-Ong’

**Figure 2:**
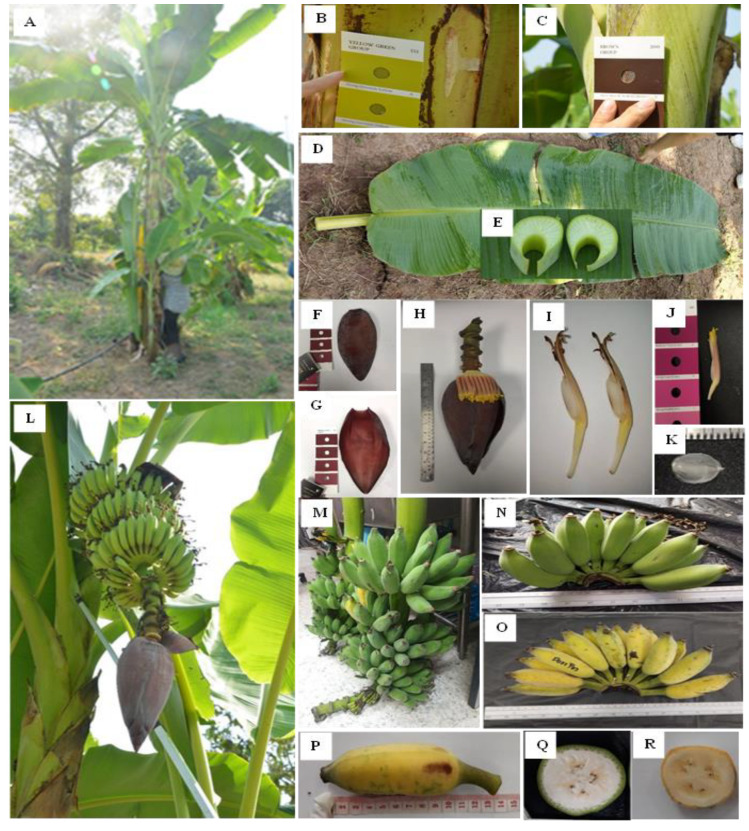
Morphological characteristics of *Musa* (ABB) ‘Mali-Ong’. (**A**) Banana plant; (**B**) pseudostem color; (**C**) leaf blotches at petiole base; (**D**) leaf; (**E**) petiole canal leaf III; (**F**) color of bract external face; (**G**) color of bract internal face; (**H**) male bud; (**I**) male flower; (**J**) compound tepal color; (**K**) free tepal; (**L**) inflorescence; (**M**) bunch shape; (**N**) hand before maturity; (**O**) hand at maturity; (**P**) fruit at maturity; (**Q**) color of pulp before maturity; and (**R**) color of pulp at maturity.

The 77 morphological features are listed in Table 4 and shown in Figure 2. An intermediate leaf habit characterizes the overall appearance of a plant. ‘Nam Wa Mali-Ong’ has a tall pseudostem with an average height (326.8225 cm) > 3 m. The pseudostem is slender (60.767 cm), yellow-green, and waxy. There are eight suckers between a quarter and three-quarters of the height of the parent plant. The petiole base leaf blotches are small, brown, with a straight canal with erect margins. The petiole margin width is 1.5–2.0 cm, and the petiole is relatively short (44–55.50 cm). 

The leaf blade length is 199–223.5 cm, the width is 58–81.5 cm, and the leaf ratio is 2.7. The shape of the leaf blade base is rounded on both sides. The upper surface of the leaf is shiny and very waxy. The lower surface of the leaf is yellow-green, with a dull appearance. The leaf midrib ventral and dorsal surfaces are yellow-green, with symmetric leaf blade insertion sites on the petiole. 

The peduncle is medium length (39 cm), 3.8 cm in width, yellow-green, and hairless. The bunch hangs at a slight angle; it has a cylindrical shape and a very compact appearance. The male bud shape is ovoid. The male flower is yellow-orange. The male bud length at harvest is 27–29 cm. Bract base shape: tiny shoulder, obtuse apex, dark red exterior face, dark red interior face, deep grooves, and a waxy texture. Before falling, the bract rolls and creates very prominent scars on the rachis. Male flowers fall after the bracts. Free tepal is oval in shape, translucent white, obtuse, and has a developed apex. There are five anthers, which are yellow with red at the lobe margin, anther exertion is at the same level, and the filament is yellow-green. The ovary shape is curved, the basic color is yellow, the style is straight, and the stigma is yellow. The arrangement of the ovules is four-rowed.

A hand contains 15–18 fruits with a 10 cm fruit length. The shape is straight (or slightly curved). The transverse section of the fruit is round. The fruit apex is bottle-necked. The persistent style is the remains of the flower relicts at the fruit apex. The fruit pedicle length is 20 mm, the fruit pedicle width is 10 mm, and the pedicle surface is hairless. The color of the immature fruit peel is bright yellow-green. The mature fruit peel color is yellow. The fruit peel thickness is 2 mm. The pulp color before maturity is pale yellow. The flesh texture is firm. The predominant taste is astringent. The pulp color at maturity is yellow. The flesh texture is soft. The mature pulp is seedless, and the dominant taste is sugary.

The tallest plants with the largest leaves were observed in clone A. Height (368.5 ± 9.2 cm), pseudostem circumference (68.5 ± 3.5 cm), petiole length (51.5 ± 0.7 cm.), leaf blade length (223.5 ± 14.9 cm), and leaf blade width (81.5 ± 2.1 cm) are presented in Table 5. When six quantitative characteristics were evaluated, only the leaf blade width was statistically significant (*p* ≤ 0.05) (Table 5). Two agronomic characteristics were observed: the number of hands per bunch and the weight per bunch. Clone I had the highest number of hands (12.0 ± 2.03) per bunch and the greatest weight per bunch (17.0 ± 2.8 kg) followed by clones D, A, and B. 

### 3.2. Genetic Variation Using Molecular Markers

#### 3.2.1. ISSR-PCR

**Figure 3:**
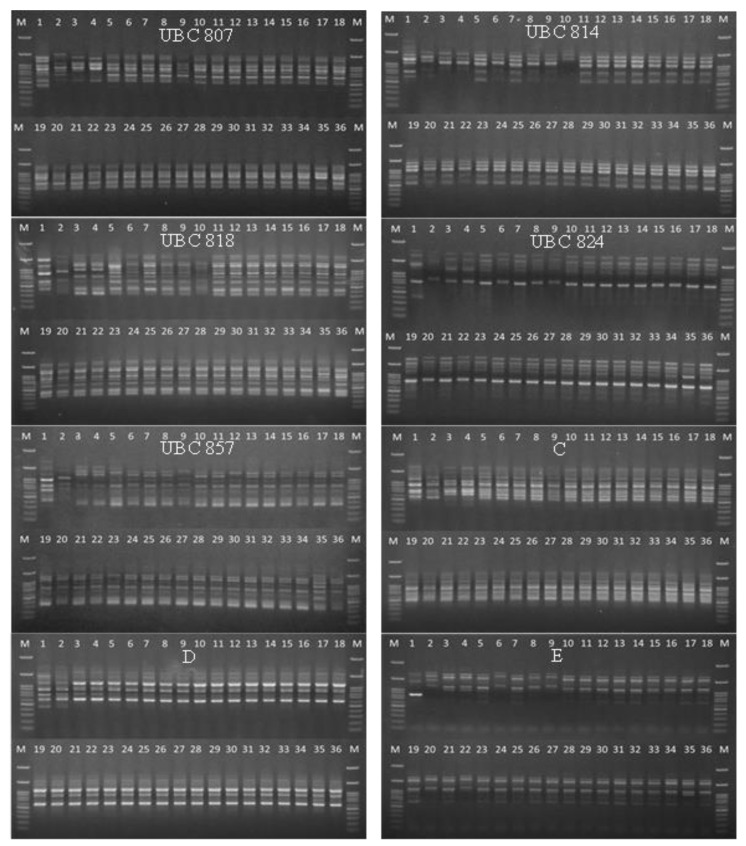
Amplification profiles of 36 banana samples using eight ISSR primers (UBC807, UBC814, UBC818, UBC824, UBC857, C, D, and E). M = 100 bp DNA ladder. Sample order (1–36 from left to right): 1= HCP; 2 = HC; 3 = TD; 4 = TNRT; 5 = NL; 6 = NU; 7 = NKK; 8 = NNJ; 9 = NPC50; 10 = NY; 11 = A021P01; 12 = A021P08; 13 = A011P08; 14 = A011P08; 15 = B091P04; 16 = B091P04; 17 = C041P03; 18 = C041P06; 19 = C051P06; 20 = C061P09; 21 = D071P03; 22 = D071P07; 23 = D081P05; 24 = D081P08; 25 = E031P01; 26 = 031P03; 27 = F061P03; 28 = F061P08; 29 = G121P03; 30 = G121P05; 31 = H101P03; 32 = H101P07; 33 = I11P04; 34 = I 111P08; 35 = P131P03; 36 = P131.

The except for primer E exhibited a monomorphic pattern, and the other primers (UBC-807, UBC-814, UBC-818, UBC-824, UBC-857, C, and D) produced polymorphic banding patterns (Figure 3). A total of 76 alleles and 62 polymorphic bands were observed. The alleles ranged from 350 to 2500 bp, with an average of 9.5 alleles per primer and an 80% polymorphism rate. The primer UBC-818 produced 13 polymorphic bands with a polymorphic percentage of 100%. The PIC values varied from 0.2550 to 0.4990. Thirty-six samples of *Musa* (AA, BB, and ABB) were analyzed using primer UBC-814 (Table 6).

#### 3.2.2. SRAP-PCR

**Figure 4:**
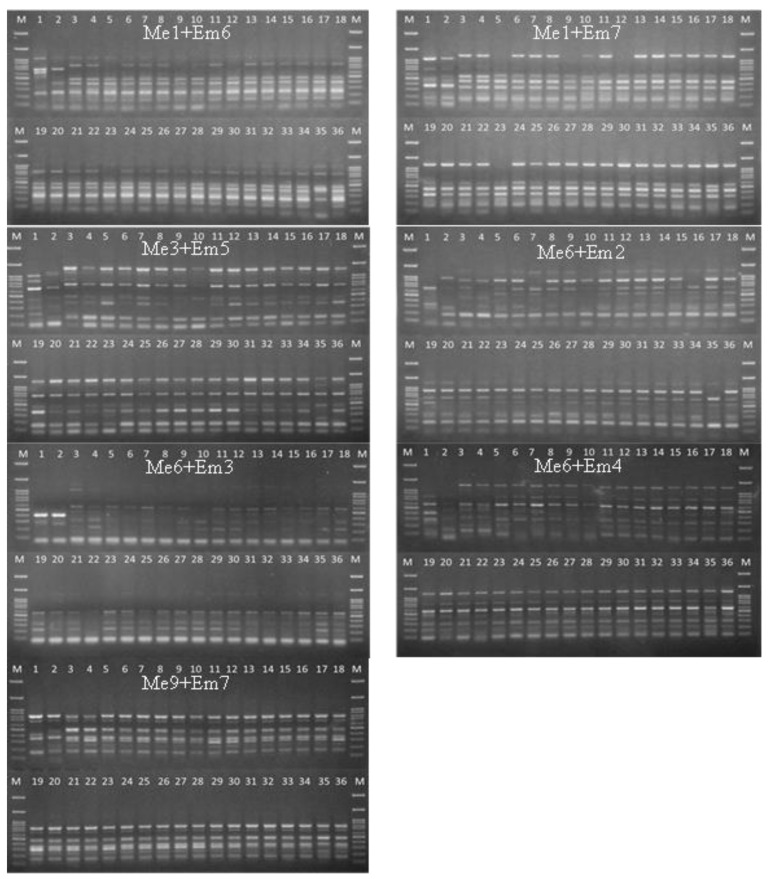
Amplification profiles of 36 banana samples using seven pairs of SRAP primers (Me1 + Em6, Me1 + Em7, Me3 + Em5, Me6 + Em2, Me6 + Em3, Me6 + Em4, and Me9 + Em7). M = 100 bp DNA ladder. Sample order (1–36 from left to right): 1 = HCP; 2 = HC; 3 = TD; 4 = TNRT; 5 = NL; 6 = NU; 7 = NKK; 8 = NNJ; 9 = NPC50; 10 = NY; 11 = A021P01; 12 = A021P08; 13 = A011P08; 14 = A011P08; 15 = B091P04; 16 = B091P04; 17 = C041P03; 18 = C041P06; 19 = C051P06; 20 = C061P09; 21 = D071P03; 22 = D071P07; 23 = D081P05; 24 = D081P08; 25 = E031P01; 26 = 031P03; 27 = F061P03; 28 = F061P08; 29 = G121P03; 30 = G121P05; 31 = H101P03; 32 = H101P07; 33 = I11P04; 34 = I 111P08; 35 = P131P03; 36 = P131P.

Of the 64 SRAP primer pairs, seven revealed distinct DNA bands. The SRAP primer sets (Table 3) yielded an average of nine fragments per primer combination, resulting in 65 bands for detecting genetic diversity (Table 7). A total of 65 bands were assessed, of which 57 (or 87%) were polymorphic. Alleles ranged from 100 to 1300 bp (Figure 4), and PIC values ranged between 0.2822 and 0.5.

### 3.3. Cluster Analysis

**Figure 5:**
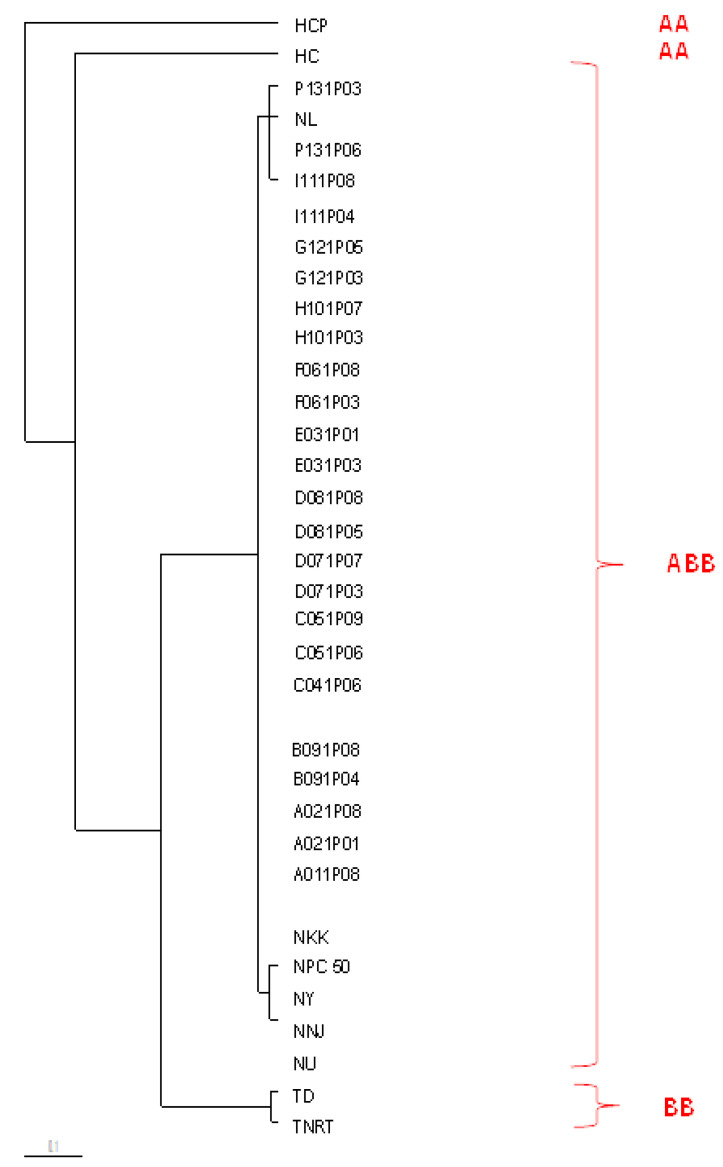
Dendrogram constructed using the unweighted pair-group method with arithmetic average (UPGMA) analysis based on ISSR similarity coefficients of 36 *Musa* samples.

**Figure 6:**
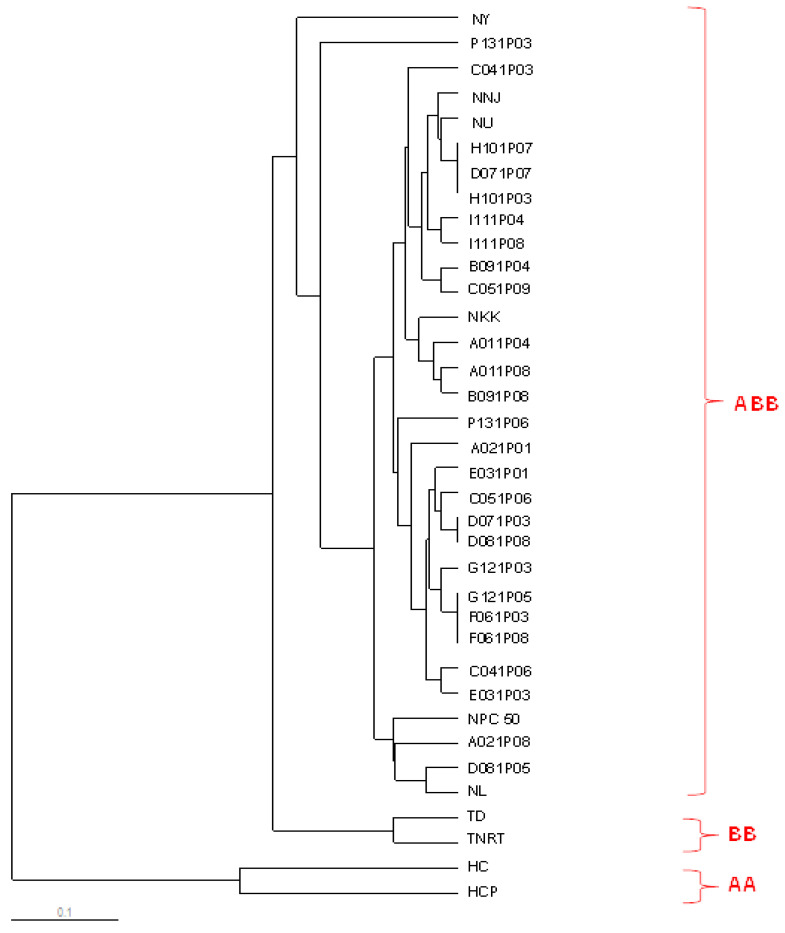
Dendrogram constructed using UPGMA analysis based on SRAP similarity coefficients of 36 *Musa* samples.

The ISSR similarity coefficient varied from 0.11 to 1.00 in the 36 samples. The coefficient of similarity between samples AA (‘Kluai Hom Champa’) and BB (‘Kluai Tani Dum’) was 0.11, and that between ABB samples (‘Kluai Nam Wa Mali-Ong’ and ‘Kluai Nam Wa Kabkhao’) and AA (‘Kluai Hom Champa’) was 0.12. The ABB samples (‘Kluai Nam Wa Mali-Ong’ and ‘Kluai Nam Wa Kabkhao’) shared a high similarity coefficient (0.91–1.00) with the ABB ‘Nam Wa’ sample. The ISSR cluster analysis divided the 36 accessions into two distinct clusters based on the similarity coefficient (Table 8). Cluster I contained only *Musa* ‘Hom Champa’ (AA), whereas Cluster II was divisible into two subgroups. Subgroup A included only *Musa* ‘Hom Chan’ (AA); subgroup B contained all ABB samples, including ‘Nam Wa Looksaileung’, Kabkhoa, Ubon, Pakchong 50, Nuanchan, and Yak. *Musa* BB included ‘Kluai Tani Dum’ and ‘Kluai Tani Nakhon Si Thammarat’ (Figure 5). SRAP data for the 36 samples revealed similarity coefficients ranging from 0.06 to 1.00. Compared with the ‘Hom Champa’ and ‘Kluai Nam Wa Mali-Ong’ samples, the other samples showed a lower similarity coefficient (0.06). Figure 6 shows that the dendrogram produced using SRAP markers contains two major clusters based on similarity coefficients (Table 9). The first cluster includes the ABB and BB genome accessions. The second cluster is entirely composed of AA accessions. SRAP markers clearly distinguished between the AA, BB, and ABB genomic groups and generated a higher percentage of polymorphism (87%) than ISSR markers (81.6%). However, SRAP and ISSR markers placed all the ABB accessions in the same cluster.

## 4. Discussion

*Musa* (ABB) ‘Mali-Ong’ is a triploid that belongs to the subgroup Pisang Awak. The fruits are used for dehydrated banana processing in the northern region of Thailand. The high demand for ‘Nam Wa Mali-Ong’ in northern Thailand exceeds supply. The cultivar has been replaced with other high-yielding cultivars from different parts of Thailand or neighboring countries. However, mixing *Musa* (ABB) cultivars is a significant problem in terms of quality for the dried banana industry. Seventy-five morphological characteristics were used to describe and characterize ‘Nam Wa Mali-Ong’. The general characteristics of ‘Nam Wa Mali-Ong’ are comparable to those of other ABB species, including ‘Nam Wa Looksaileung and Kabkhoa. ‘Nam Wa Mali-Ong’ has a thinner pseudostem, which has a height >3. The base of the leaf blade is rounded on both sides, with the upper surface being green and the lower surface yellow-green and extremely waxy. The peduncle is medium in length, yellow-green, and hairless. The ovule consists of four rows. Before maturation, the pulp is pale yellow; however, after maturation, it becomes yellow. These cultivars can be distinguished by the ripe pulp color; ‘Nam Wa Looksaileung’ is bright yellow, Kabkhoa is pale pink to white, and Mali Ong is yellow. The leaf blade width was the only quantitative attribute that was statistically distinct, and this distinction may have resulted from an epistatic or pleiotropic interaction [22]. The growth and yield of eight clones, including ‘Nam Wa Pak Chong 50’, ‘Nam Wa Mali Ong’, ‘Nam Wa Tanao Si’, ‘Nam Wa Ngoen’, ‘Nam Wa Khom’, ‘Nam Wa Thong Ma Eng’, and ‘Nam Wa Dam’, were documented [23]. The heights of the pseudostems of the eight clones ranged from 2.7 to 4.1 m. The pedicle length and peel thickness of distinct clones varied. Bunch weights ranged from 19 to 31 kg. Fruit was harvested 58–64 weeks after the planting date. ‘Nam Wa Mali-Ong’ was reported as having low bunch weights [23]. In this study, the first crop cycle of ‘Nam Wa Mali Ong’ yielded bunch weights of 12.17 to 17.00 kg. Bunch weight yield often increases between the second and third crop cycles [24]. The breadth of the leaf blades was observed to vary substantially. Clone D had the shortest leaf blade size; however, this did not affect the yield per bunch. This investigation clarified 77 morphological ‘Nam Wa Mali Ong’ characteristics.

The ISSR and SRAP markers showed high PIC values among the *Musa* accessions, ranging from 0.2550 to 0.4998 (average, 0.3850; Table 4) and from 0.2822 to 0.5 (average, 0.4527; Table 5), respectively. The PIC values indicated that ISSR and SRAP markers effectively revealed genetic variation among the banana cultivars. Compared with AFLP markers, SRAP markers provide three times more specific and unique bands [25]. According to a previous study, *Musa* (ABB) showed low variation when the genetic relationship was assessed using SSR [2]. SRAP markers are recommended as a more efficient means of distinguishing between *Musa schizocarpa*, *M. balbisiana*, and *M. acuminata* in the Eumusa section and between plantains and cooking bananas among triploid cultivars [26]. Previous research using ISSR and SCoT markers to investigate the genetic diversity of *Musa* species with different genomes showed that ISSR markers produced lower levels of genetic polymorphism [27]. Phitsanulok Province is in the low northern region of Thailand, comprising nine districts, Muang, Bang Rakam, Bang Kratum, Nakhon Thai, Chat Trakarn, Wang Thong, Neon-Maprang, Wat Bot, and Prom Phiram. Plains and plateaus dominate the geography of Phitsanulok Province. The highest point, Chart Trakan, is 800 m above sea level. The genetic variation of the accessions was not affected by the environment. Unlike morphological markers, molecular markers are unaffected by environmental factors [28]. The morphological characteristics and flowering of the ABB, BB, and AA groups require 12–16 months to be observed, and bunching occurs yearly. Environmental parameters regularly play a strong role in morphological expressions in plants, which occasionally may not correlate with molecular markers because of modifications in non-coding sequences. The leaf blade width separated the nine clones into four groups. The clones A, B, D, and I can be developed to produce an elite high yield cultivar that can be used in the processed food industry in the northern region of Thailand. Substitution of other high bunch yield ABB cultivars for ‘Nam Wa Mali-Ong’ may lead to its disappearance from fields. The findings of this study support that ex situ germplasm conservation in the northern region of Thailand should be urgently conducted on-farm and in vitro for the sustainable use of ‘Nam Wa Mali Ong’ in the processed food industry in Thailand. Novel Mali-Ong plants that are tolerant to diseases such as yellow sigatoka, anthracnose, and brown spot, which are destroying banana farms and reducing bunch yields, also need to be developed in the future. ISSR and SRAP markers effectively classified *Musa* AA, BB, and ABB cultivars. The data from the SRAP and ISSR markers and morphological characteristics produced corresponding results regarding the variation in Nam Wa Mali Ong.

## 5. Conclusions

This study described 77 morphological traits of *Musa* (ABB) ‘Mali-Ong’. Clones A, B, D, and I appeared to be superior with the highest bunch weight among the collected clones. SRAP markers were used to detect DNA variation in *Musa* AA, ABB, and BB groups. The ABB accessions were found to have a higher degree of genetic similarity and were clustered together. SRAP and ISSR makers can be employed to conserve and breed bananas.

## Figures and Tables

**Table 1:** List of *Musa* (ABB) ‘Mali-Ong’ samples collected from Phitsanulok Province, Thailand, and reference genomes AA and BB.

No.	Local Name	Genomic Group	Collection Site	Code
1.	‘Kluai Hom Champa’	AA	Phitsanulok	HCP
2.	‘Kluai Hom Chan’	AA	Sukhothai Horticultural Research Center	HC
3.	‘Kluai Tani Dam’	BB	Phitsanulok	TD
4.	‘Kluai Tani Nakhon Si Thammarat’	BB	Sukhothai Horticultural Research Center	TNRT
5.	‘Kluai Nam Wa Looksileuang’	ABB	Phitsanulok	NYL
6.	‘Kluai Nam Wa Ubon	ABB	Phitsanulok	NU
7.	‘Kluai Nam Wa Kabkhao	ABB	Phitsanulok	NKK
8.	‘Kluai Nam Wa Nuanchan	ABB	Phitsanulok	NNJ
9.	‘Kluai Nam Wa Pakchong 50	ABB	Research Center (Pakchong)	NPC 50
10.	‘Kluai Nam Wa Yak	ABB	Research Center (Pakchong)	NY
11.	‘Kluai Nam Wa Mali-Ong’	ABB	Mueang	A021P01
12.	‘Kluai Nam Wa Mali-Ong’	ABB	Mueang	A021P08
13.	‘Kluai Nam Wa Mali-Ong’	ABB	Mueang	A011P04
14.	‘Kluai Nam Wa Mali-Ong’	ABB	Mueang	A011P08
15.	‘Kluai Nam Wa Mali-Ong’	ABB	Wang Thong	B091P04
16.	‘Kluai Nam Wa Mali-Ong’	ABB	Wang Thong	B091P08
17.	‘Kluai Nam Wa Mali-Ong’	ABB	Bang Rakam	C041P03
18.	‘Kluai Nam Wa Mali-Ong’	ABB	Bang Rakam	C041P06
19.	‘Kluai Nam Wa Mali-Ong’	ABB	Bang Rakam	C051P06
20.	‘Kluai Nam Wa Mali-Ong’	ABB	Bang Rakam	C061P09
21.	‘Kluai Nam Wa Mali-Ong’	ABB	Wat Bot	D071P03
22.	‘Kluai Nam Wa Mali-Ong’	ABB	Wat Bot	D071P07
23.	‘Kluai Nam Wa Mali-Ong’	ABB	Wat Bot	D081P05
24.	‘Kluai Nam Wa Mali-Ong’	ABB	Wat Bot	D081P08
25.	‘Kluai Nam Wa Mali-Ong’	ABB	Bang Krathum	E031P01
26.	‘Kluai Nam Wa Mali-Ong’	ABB	Bang Krathum	E031P03
27.	‘Kluai Nam Wa Mali-Ong’	ABB	Phrom Phiram	F061P03
28.	‘Kluai Nam Wa Mali-Ong’	ABB	Phrom Phiram	F061P08
29.	‘Kluai Nam Wa Mali-Ong’	ABB	Noen Maprang	G121P03
30.	‘Kluai Nam Wa Mali-Ong’	ABB	Noen Maprang	G121P05
31.	‘Kluai Nam Wa Mali-Ong’	ABB	Nakhon Thai	H101P03
32.	‘Kluai Nam Wa Mali-Ong’	ABB	Nakhon Thai	H101P07
33.	‘Kluai Nam Wa Mali-Ong’	ABB	Chat Trakan	I111P04
34.	‘Kluai Nam Wa Mali-Ong’	ABB	Chat Trakan	I111P08
35.	‘Kluai Nam Wa Mali-Ong’	ABB	Pak Chong, Nakhon Ratchasima	P131P03
36.	‘Kluai Nam Wa Mali-Ong’	ABB	Pak Chong, Nakhon Ratchasima	P131P06

**Table 2:** Inter simple sequence repeat (ISSR) primers showing strong amplification and good reproducibility selected in this study.

Primer Name	Primer Nucleotide Sequence (5′-3′)	GC	Tm	Source
UBC-807	AGAGAGAGAGAGAGAGT	47	52	[11]
UBC-814	CTCTCTCTCTCTCTCTA	47	50	[11]
UBC-818	CACACACACACACACAG	53	50	[11]
UBC-824	TCTCTCTCTCTCTCTCG	53	50	[15]
UBC-857	ACACACACACACACACVG	50	50	[16]
C	GAGGAGGAGGAGGAGAC	65	52	[17,18,19]
D	GAGGAGGAGGAGGAGAT	59	50	[17,18,19]
E	GGGGTGGGGTGGGGT	80	50	[18,19]

**Table 3:** Forward and reverse sequence-related amplified polymorphism (SRAP) primers and seven primer combinations applied in this study.

Primer Information	
Forward primer	
Me 1	TGAGTCCAAACCGGATA
Me3	TGAGTCCAAACCGGAAT
Me6	TGAGTCCAAACCGGTAG
Me9	TGAGTCCAAACCGGTCA
Reverse primer	
Em2	GACTGCGTACGAATTTGC
Em3	GACTGCGTACGAATTGAC
Em4	GACTGCGTACGAATTTGA
Em6	GACTGCGTACGAATTGCA
Em7	GACTGCGTACGAATTATG
Primer combination	
Me1 + Em6	
Me1 + Em7	
Me3 + Em5	
Me6 + Em2	
Me6 + Em3	
Me6 + Em4	
Me9 + Em7	

**Table 4:** Morphological characteristics of *Musa* (ABB) ‘Mali-Ong’. The evaluation was performed in Phitsanulok Province, Thailand using banana descriptors [14]. Color was determined using a Royal Horticultural Society (RHS) color chart.

No.	Morphological Characteristics	Description
1	Leaf habit	Intermediate, with normal leaves
2–6	Pseudostem	Average height, 326.77 ± 22.45 cm; average pseudostem circumference, 60.72 ± 6.7 cm; slender; color, yellow-green group 144 C; waxy
7–9	Number of suckers	Eight suckers, close to parent, between ¼ and ¾ of the height of the parent plant
10–11	Leaf blotches at petiole base	Small blotches, color of blotches; brown group 200 A
12	Petiole canal leaf III	Straight with erect margins
13	Petiole margin color	Brown group 200 A
14	Petiole margin width	1.5–2 cm
15	Petiole length	44–55.50 cm (short)
16	Leaf blade length	199–223.5 cm
17	Leaf blade width	58.5–81.5 cm
18	Color of leaf upper surface	Green group 137 B
19	Appearance of leaf upper surface	Shiny
20	Color of leaf lower surface	Yellow-green group 148B
21	Appearance of leaf lower surface	Dull
22	Wax on leaves	Very waxy
23	Insertion points of leaf blades on petiole	Symmetric
24	Shape of leaf blade base	Both sides rounded
25	Color of midrib ventral surface	Yellow-green group N144D
26	Color of midrib dorsal surface	Yellow-green group 154 D
27–29	Inflorescence/male bud	Medium peduncle length (39 cm), peduncle width 3.8 cm, peduncle color strong yellow–green 144D
30	Peduncle hairiness	Peduncle hairless
31	Bunch position	Slightly angle
32	Bunch shape	Cylindrical
33	Bunch appearance	Very compact
34	Male bud size	Length of male bud at harvest, 27–29 cm
35	Bract base shape	Small shoulder
36	Bract apex shape	Obtuse
37	Color of bract external face	Dark red, greyed-purple group 183 A
38	Color of bract internal face	Dark red, greyed-purple group 183 B
39	Bract scars on rachis	Very prominent
40	Bract behavior before falling	Rolling
41	Wax on bract	Very waxy
42	Presence of grooves on the bract	Strong grooved
43	Male flower/male flower behavior	Falling after the bract
44	Compound tepal basic color	Red-purple 60A
45	Lobe color of compound tepal	Yellow 13 A
46	Free tepal color	Translucent white
47	Free tepal shape	Oval
48	Free tepal apex development	Developed
49	Free tepal apex shape	Obtuse
50	Anther exertion	In relation to the base of the lobes on the compound tepal same level
51	Filament color	Yellow-green 11 D
52	Anther color	Yellow group 11 B with Red 39 B at margin
53	Style basic color	Yellow group 11 D
54	Style shape	Straight
55	Color of stigma	Yellow group 11 B
56	Ovary shape	Curved
57	Ovary basic color	Yellow group 11 C
58	Arrangement of ovules	Four-rowed
59	Fruit/number of Fruits	15–18
60	Fruit length	10 cm
61	Fruit shape	Straight (or slightly curved)
62	Transverse section of fruit	Round
63	Fruit apex	Bottle-necked
64	Remains of flower relicts at fruit apex	Persistent style
65	Fruit pedicle length	20 mm
66	Fruit pedicle width	10 mm
67	Pedicle surface	Hairless
68	Immature fruit peel color	Strong yellow-green B
69	Mature fruit peel color	Yellow group 9 B
70	Fruit peel thickness	2 mm
71	Pulp color before maturity	Yellow group 11D
72	Flesh texture	Firm
73	Predominant taste	Astringent
74	Pulp color at maturity	Yellow group 8D
75	Flesh texture	Soft
76	Predominant taste	Sugary
77	Presence of seed with source of pollen	Seedless

**Table 5:** Quantitative characteristics of *Musa (ABB)* ‘Mali-Ong’ collected from nine farms.

Clone	Morphological Characteristic							
	Pseudostem		Petiole		Leaf Blade			
	Height (cm)	Circumference (cm)	Margin Width (cm)	Length (cm)	Length (cm)	Width (cm)	No. of Hand/Bunch	Weight/Bunch (kg)
A	368.5 ± 9.2	68.5 ± 3.5	2.0 ± 0.0	51.5 ± 0.7	223.5 ± 14.9	81.5 ± 2.1 ^a^	10.67 ± 0.0 ^abc^	14.67 ± 1.4 ^ab^
B	362.5 ± 17.7	67.5 ± 2.1	2.0 ± 0.0	49.5 ± 0.7	199 ± 2.8	80.0 ± 1.4 ^ab^	9.0 ± 0.7 ^abc^	13.67 ± 0.7 ^ab^
C	323.5 ± 26.2	49.0 ± 2.8	1.5 ± 0.7	44.0 ± 4.2	175.5 ± 19.1	58.5 ± 5.7 ^d^	8.0 ± 0.0 ^c^	12.17 ± 0.4 ^b^
D	311.5 ± 26.2	59.5 ± 3.5	1.75 ± 0.4	55.5 ± 2.1	183.5 ± 29.0	59.0 ± 5.7 ^d^	11.7 ± 0.7 ^ab^	15.7 ± 0.7 ^ab^
E	320.0 ± 28.3	63.5 ± 3.5	2.15 ± 0.2	47.5 ± 6.4	213.5 ± 10.6	74.5 ± 3.5 ^abc^	8.67 ± 1.4 ^bc^	12± 2.8 ^b^
F	282.5 ± 53,0	53.0 ± 12.7	2.0 ± 0.0	55.5 ± 6.4	181 ± 33.9	66.8 ± 8.1 ^cd^	8 ± 0.0 ^c^	12.2 ± 0.0 ^b^
G	315.0 ± 26.9	56.25 ± 3.9	1.75 ± 0.4	51.5 ± 2.1	202.5 ± 27.6	64.8 ± 7.4 ^cd^	8.7 ± 1.4 ^bc^	12.7 ± 1.4 ^b^
H	314.0 ± 2.8	61.5 ± 0.7	1.8 ± 0.4	54.5 ± 10.6	199.5 ± 0.7	69.5 ± 0.7 ^abc^	8.0 ± 0.0 ^c^	12.2 ± 0.0 ^b^
I	343.5 ± 12.0	67.8 ± 2.5	2.0 ± 0.0	49.5 ± 0.7	212.5 ± 20.5	72.8 ± 1.1 ^abc^	12.0 ± 2.0 ^a^	17.0 ± 2.8 ^a^
	ns	ns	ns	ns	ns	*	*	*

Data are mean values ± standard deviation of three biological replicate of each clone. Lowercase letters are valid for the same column. ANOVA was used to test for significance, and significant differences between means were tested by DMRT and are indicated by different letters (*p* ≤ 0.05). ns = non-significant and * = significant.

**Table 6:** Polymorphism obtained from eight ISSR primers among 36 *Musa* samples.

No.	ISSR Primer	Allele Size Range (bp)	Amplified Bands			PIC Value
			Total	Polymorphic	Polymorphism (%)	
1.	UBC-807	450–1500	11	9	81.81	0.4662
2.	UBC-814	550–2500	9	7	77.78	0.4998
3.	UBC-818	350–1800	13	13	100	0.3200
4.	UBC-824	400–2300	9	9	100	0.4968
5.	UBC-857	350–2300	10	8	80	0.4278
6.	C	550–1700	10	6	60	0.3318
7.	D	450–1600	7	3	42.86	0.2550
8.	E	650–1700	7	7	100	0.2822
Total			76	62		3.0796
Average			9.5	7.75	80.31	0.3850

PIC—polymorphic information content.

**Table 7:** Polymorphism obtained from seven SRAP primer combinations among 36 *Musa* samples.

No.	SRAP Primer	Allele Size Range (bp)	Amplified Bands			PIC Value
			Total	Polymorphic	Polymorphism (%)	
1.	Me1 + Em6	150–1100	11	10	90.9	0.4992
2.	Me1 + Em7	100–1200	9	8	88.88	0.4928
3.	Me3 + Em5	150–1300	12	11	91.66	0.4838
4.	Me6 + Em2	200–1300	8	8	100	0.2822
5.	Me6 + Em3	250–1300	8	8	100	0.4352
6.	Me6 + Em4	100–1300	10	8	80	0.4758
7.	Me9 + Em7	150–800	7	4	57.14	0.5000
Total			65	57		3.1690
Average			9.29	8.14	86.94	0.4527

**Table 8:** Similarity coefficient matrix of 36 *Musa* samples based on ISSR primers.

	HJP	HCH	TD	TNRT	NLY	NU	NKK	NNJ	NPC50	NVY	A11	A12	A21	A22	B11	B12	C11	C12	C21	C22	D11	D12	D21	D22	E11	E12	F11	F12	G11	G12	H11	H12	I11	I12	CT1	CT2
HJP	1.00																																			
HCH	0.29	1.00																																		
TD	0.11	0.33	1.00																																	
TNRT	0.10	0.35	0.97	1.00																																
NLY	0.10	0.28	0.60	0.62	1.00																															
NU	0.14	0.33	0.63	0.64	0.91	1.00																														
NKK	0.12	0.30	0.58	0.60	0.97	0.94	1.00																													
NNJ	0.14	0.33	0.63	0.64	0.91	1.00	0.94	1.00																												
NPC50	0.12	0.31	0.65	0.67	0.87	0.97	0.90	0.97	1.00																											
NVY	0.14	0.33	0.63	0.64	0.91	1.00	0.94	1.00	0.97	1.00																										
A11	0.12	0.30	0.58	0.60	0.97	0.94	1.00	0.94	0.90	0.94	1.00																									
A12	0.12	0.30	0.58	0.60	0.97	0.94	1.00	0.94	0.90	0.94	1.00	1.00																								
A21	0.12	0.30	0.58	0.60	0.97	0.94	1.00	0.94	0.90	0.94	1.00	1.00	1.00																							
A22	0.12	0.30	0.58	0.60	0.97	0.94	1.00	0.94	0.90	0.94	1.00	1.00	1.00	1.00																						
B11	0.12	0.30	0.58	0.60	0.97	0.94	1.00	0.94	0.90	0.94	1.00	1.00	1.00	1.00	1.00																					
B12	0.12	0.30	0.58	0.60	0.97	0.94	1.00	0.94	0.90	0.94	1.00	1.00	1.00	1.00	1.00	1.00																				
C11	0.12	0.30	0.58	0.60	0.97	0.94	1.00	0.94	0.90	0.94	1.00	1.00	1.00	1.00	1.00	1.00	1.00																			
C12	0.12	0.30	0.58	0.60	0.97	0.94	1.00	0.94	0.90	0.94	1.00	1.00	1.00	1.00	1.00	1.00	1.00	1.00																		
C21	0.12	0.30	0.58	0.60	0.97	0.94	1.00	0.94	0.90	0.94	1.00	1.00	1.00	1.00	1.00	1.00	1.00	1.00	1.00																	
C22	0.12	0.30	0.58	0.60	0.97	0.94	1.00	0.94	0.90	0.94	1.00	1.00	1.00	1.00	1.00	1.00	1.00	1.00	1.00	1.00																
D11	0.12	0.30	0.58	0.60	0.97	0.94	1.00	0.94	0.90	0.94	1.00	1.00	1.00	1.00	1.00	1.00	1.00	1.00	1.00	1.00	1.00															
D12	0.12	0.30	0.58	0.60	0.97	0.94	1.00	0.94	0.90	0.94	1.00	1.00	1.00	1.00	1.00	1.00	1.00	1.00	1.00	1.00	1.00	1.00														
D21	0.12	0.30	0.58	0.60	0.97	0.94	1.00	0.94	0.90	0.94	1.00	1.00	1.00	1.00	1.00	1.00	1.00	1.00	1.00	1.00	1.00	1.00	1.00													
D22	0.12	0.30	0.58	0.60	0.97	0.94	1.00	0.94	0.90	0.94	1.00	1.00	1.00	1.00	1.00	1.00	1.00	1.00	1.00	1.00	1.00	1.00	1.00	1.00												
E11	0.12	0.30	0.58	0.60	0.97	0.94	1.00	0.94	0.90	0.94	1.00	1.00	1.00	1.00	1.00	1.00	1.00	1.00	1.00	1.00	1.00	1.00	1.00	1.00	1.00											
E12	0.12	0.30	0.58	0.60	0.97	0.94	1.00	0.94	0.90	0.94	1.00	1.00	1.00	1.00	1.00	1.00	1.00	1.00	1.00	1.00	1.00	1.00	1.00	1.00	1.00	1.00										
F11	0.12	0.30	0.58	0.60	0.97	0.94	1.00	0.94	0.90	0.94	1.00	1.00	1.00	1.00	1.00	1.00	1.00	1.00	1.00	1.00	1.00	1.00	1.00	1.00	1.00	1.00	1.00									
F12	0.12	0.30	0.58	0.60	0.97	0.94	1.00	0.94	0.90	0.94	1.00	1.00	1.00	1.00	1.00	1.00	1.00	1.00	1.00	1.00	1.00	1.00	1.00	1.00	1.00	1.00	1.00	1.00								
G11	0.12	0.30	0.58	0.60	0.97	0.94	1.00	0.94	0.90	0.94	1.00	1.00	1.00	1.00	1.00	1.00	1.00	1.00	1.00	1.00	1.00	1.00	1.00	1.00	1.00	1.00	1.00	1.00	1.00							
G12	0.12	0.30	0.58	0.60	0.97	0.94	1.00	0.94	0.90	0.94	1.00	1.00	1.00	1.00	1.00	1.00	1.00	1.00	1.00	1.00	1.00	1.00	1.00	1.00	1.00	1.00	1.00	1.00	1.00	1.00						
H11	0.12	0.30	0.58	0.60	0.97	0.94	1.00	0.94	0.90	0.94	1.00	1.00	1.00	1.00	1.00	1.00	1.00	1.00	1.00	1.00	1.00	1.00	1.00	1.00	1.00	1.00	1.00	1.00	1.00	1.00	1.00					
H12	0.12	0.30	0.58	0.60	0.97	0.94	1.00	0.94	0.90	0.94	1.00	1.00	1.00	1.00	1.00	1.00	1.00	1.00	1.00	1.00	1.00	1.00	1.00	1.00	1.00	1.00	1.00	1.00	1.00	1.00	1.00	1.00				
I11	0.12	0.30	0.58	0.60	0.97	0.94	1.00	0.94	0.90	0.94	1.00	1.00	1.00	1.00	1.00	1.00	1.00	1.00	1.00	1.00	1.00	1.00	1.00	1.00	1.00	1.00	1.00	1.00	1.00	1.00	1.00	1.00	1.00			
I12	0.12	0.30	0.58	0.60	0.97	0.94	1.00	0.94	0.90	0.94	1.00	1.00	1.00	1.00	1.00	1.00	1.00	1.00	1.00	1.00	1.00	1.00	1.00	1.00	1.00	1.00	1.00	1.00	1.00	1.00	1.00	1.00	1.00	1.00		
CT1	0.13	0.28	0.60	0.62	0.93	0.91	0.97	0.91	0.87	0.91	0.97	0.97	0.97	0.97	0.97	0.97	0.97	0.97	0.97	0.97	0.97	0.97	0.97	0.97	0.97	0.97	0.97	0.97	0.97	0.97	0.97	0.97	0.97	0.97	1.00	
CT2	0.12	0.30	0.58	0.60	0.97	0.94	1.00	0.94	0.90	0.94	1.00	1.00	1.00	1.00	1.00	1.00	1.00	1.00	1.00	1.00	1.00	1.00	1.00	1.00	1.00	1.00	1.00	1.00	1.00	1.00	1.00	1.00	1.00	1.00	0.97	1.00

**Table 9:** Similarity coefficient matrix of 36 *Musa* samples based on SRAP primer combination.

	HJP	HCH	TD	TNRT	NLY	NU	NKK	NNJ	NPC50	NVY	A11	A12	A21	A22	B11	B12	C11	C12	C21	C22	D11	D12	D21	D22	E11	E12	F11	F12	G11	G12	H11	H12	I11	I12	CT1	CT2
HJP	1.00																																			
HCH	0.59	1.00																																		
TD	0.05	0.17	1.00																																	
TNRT	0.06	0.22	0.88	1.00																																
NLY	0.14	0.29	0.59	0.54	1.00																															
NU	0.12	0.26	0.69	0.63	0.88	1.00																														
NKK	0.11	0.24	0.68	0.58	0.82	0.88	1.00																													
NNJ	0.12	0.26	0.64	0.63	0.88	0.94	0.88	1.00																												
NPC50	0.13	0.28	0.67	0.66	0.85	0.85	0.79	0.85	1.00																											
NVY	0.14	0.24	0.56	0.60	0.61	0.72	0.67	0.72	0.74	1.00																										
A11	0.10	0.22	0.70	0.60	0.85	0.85	0.91	0.85	0.82	0.70	1.00																									
A12	0.10	0.22	0.70	0.60	0.85	0.79	0.85	0.79	0.88	0.65	0.88	1.00																								
A21	0.09	0.21	0.73	0.63	0.83	0.88	0.94	0.88	0.80	0.68	0.91	0.91	1.00																							
A22	0.10	0.22	0.70	0.60	0.85	0.91	0.91	0.91	0.77	0.65	0.88	0.88	0.97	1.00																						
B11	0.10	0.22	0.70	0.60	0.85	0.91	0.91	0.91	0.82	0.70	0.94	0.88	0.91	0.94	1.00																					
B12	0.11	0.24	0.68	0.58	0.82	0.88	0.94	0.88	0.74	0.62	0.85	0.85	0.94	0.97	0.91	1.00																				
C11	0.09	0.24	0.68	0.63	0.88	0.88	0.82	0.88	0.79	0.67	0.91	0.85	0.88	0.91	0.91	0.88	1.00																			
C12	0.07	0.21	0.67	0.63	0.83	0.88	0.88	0.88	0.85	0.73	0.91	0.85	0.88	0.85	0.91	0.82	0.88	1.00																		
C21	0.09	0.21	0.68	0.63	0.82	0.88	0.88	0.88	0.85	0.78	0.91	0.85	0.88	0.85	0.91	0.82	0.88	0.94	1.00																	
C22	0.09	0.24	0.68	0.63	0.88	0.94	0.88	0.94	0.85	0.72	0.91	0.85	0.88	0.91	0.97	0.88	0.94	0.94	0.94	1.00																
D11	0.08	0.22	0.70	0.65	0.85	0.91	0.91	0.91	0.88	0.75	0.94	0.88	0.91	0.88	0.94	0.85	0.91	0.97	0.97	0.97	1.00															
D12	0.11	0.25	0.66	0.61	0.91	0.97	0.91	0.97	0.82	0.69	0.88	0.82	0.91	0.94	0.94	0.91	0.91	0.91	0.91	0.97	0.94	1.00														
D21	0.12	0.26	0.64	0.58	0.94	0.82	0.82	0.82	0.91	0.66	0.91	0.91	0.82	0.79	0.85	0.77	0.88	0.88	0.88	0.88	0.91	0.85	1.00													
D22	0.08	0.22	0.70	0.65	0.85	0.91	0.91	0.91	0.88	0.75	0.94	0.88	0.91	0.88	0.94	0.85	0.91	0.97	0.97	0.97	1.00	0.94	0.91	1.00												
E11	0.09	0.24	0.68	0.63	0.82	0.88	0.88	0.88	0.85	0.78	0.91	0.85	0.88	0.85	0.91	0.82	0.88	0.94	0.94	0.94	0.97	0.91	0.88	0.97	1.00											
E12	0.06	0.20	0.70	0.65	0.80	0.85	0.85	0.85	0.83	0.71	0.88	0.82	0.85	0.82	0.88	0.79	0.85	0.97	0.91	0.91	0.94	0.88	0.85	0.94	0.91	1.00										
F11	0.07	0.21	0.73	0.68	0.83	0.88	0.88	0.88	0.85	0.73	0.91	0.85	0.88	0.85	0.91	0.82	0.88	0.94	0.94	0.94	0.97	0.91	0.88	0.97	0.94	0.97	1.00									
F12	0.07	0.21	0.73	0.68	0.83	0.88	0.88	0.88	0.85	0.73	0.91	0.85	0.88	0.85	0.91	0.82	0.88	0.94	0.94	0.94	0.97	0.91	0.88	0.97	0.94	0.97	1.00	1.00								
G11	0.08	0.22	0.70	0.65	0.85	0.85	0.85	0.85	0.82	0.70	0.94	0.82	0.85	0.82	0.88	0.79	0.91	0.91	0.91	0.91	0.94	0.88	0.91	0.94	0.91	0.94	0.97	0.97	1.00							
G12	0.07	0.21	0.73	0.68	0.83	0.88	0.88	0.88	0.85	0.73	0.91	0.85	0.88	0.85	0.91	0.82	0.88	0.94	0.94	0.94	0.97	0.91	0.88	0.97	0.94	0.97	1.00	1.00	0.97	1.00						
H11	0.11	0.25	0.66	0.61	0.91	0.97	0.91	0.97	0.82	0.69	0.88	0.82	0.91	0.94	0.94	0.91	0.91	0.91	0.91	0.97	0.94	1.00	0.85	0.94	0.91	0.88	0.91	0.91	0.88	0.91	1.00					
H12	0.11	0.25	0.66	0.61	0.91	0.97	0.91	0.97	0.82	0.69	0.88	0.82	0.91	0.94	0.94	0.91	0.91	0.91	0.91	0.97	0.94	1.00	0.85	0.94	0.91	0.88	0.91	0.91	0.88	0.91	1.00	1.00				
I11	0.09	0.24	0.68	0.63	0.88	0.94	0.88	0.94	0.79	0.67	0.85	0.85	0.94	0.97	0.91	0.94	0.94	0.88	0.88	0.94	0.91	0.97	0.82	0.91	0.88	0.85	0.88	0.88	0.85	0.88	0.97	0.97	1.00			
I12	0.08	0.22	0.65	0.60	0.85	0.91	0.85	0.91	0.77	0.65	0.82	0.82	0.91	0.94	0.88	0.91	0.91	0.91	0.85	0.91	0.88	0.94	0.79	0.88	0.85	0.88	0.85	0.85	0.82	0.85	0.94	0.94	0.97	1.00		
CT1	0.11	0.22	0.71	0.66	0.69	0.69	0.74	0.69	0.66	0.64	0.76	0.71	0.74	0.71	0.71	0.74	0.79	0.74	0.79	0.74	0.76	0.71	0.74	0.76	0.74	0.77	0.79	0.79	0.82	0.79	0.71	0.71	0.74	0.71	1.00	
CT2	0.06	0.17	0.70	0.65	0.75	0.80	0.79	0.80	0.77	0.71	0.82	0.82	0.85	0.82	0.82	0.79	0.85	0.91	0.91	0.85	0.88	0.82	0.80	0.88	0.85	0.94	0.91	0.91	0.88	0.91	0.82	0.82	0.85	0.88	0.82	1.00

## Data Availability

Not applicable.

## References

[1] FAO (2021). Banana Market Review–Preliminary Results 2020.

[2] Rotchanapreeda T., Wongniam S., Swangpol S.C., Chareonsap P.P., Sukkaewmanee N., Somana J. (2016). Development of SSR markers from *Musa balbisiana* for genetic diversity analysis among Thai bananas. Plant Syst. Evol..

[3] Ploetz R.C. (2015). Management of Fusarium wilt of banana: A review with special reference to tropical race 4. Crop Prot..

[4] Cenci A., Sardos J., Hueber Y., Martin G., Breton C., Roux N., Swennen R., Carpentier S.C., Rouard M. (2021). Unravelling the complex story of intergenomic recombination in ABB allotriploid bananas. Ann. Bot..

[5] Tongkaew P., Tohraman A., Bungaramphai R., Mitrpant C., Aydin E. (2022). Kluai Hin (*Musa sapientum* Linn.) peel as a source of functional polyphenols identified by HPLC-ESI-QTOF-MS and its potential antidiabetic function. Sci. Rep..

[6] Somsong P., Misala P., Keeratisoontornwat K., Inwan M., Hempattarasuwan P., Duangmal K. (2015). Processing of dehydrated banana (Musa ABB “Kluai Nam Wa”) and market study in Nan Province, Thailand. Acta Hortic..

[7] Arjcharoen A., Silayoi B., Wanichkul K., Apisitwanich S. (2010). Variation of B genome in *Musa* accessions and their new identifications. Agric. Nat. Resour..

[8] de Jesus O.N., Silva S.O., Amorim E.P., Ferreira C.F., de Campos J.M., Silva G.G., Figueira A. (2013). Genetic diversity and population structure of *Musa* accessions in ex situ conservation. BMC Plant Biol..

[9] Van den houwe I., Chase R., Sardos J., Ruas M., Kempenaers E., Guignon V., Massart S., Carpentier S., Panis B., Rouard M. (2020). Safeguarding and using global banana diversity: A holistic approach. CABI Agric. Biosci..

[10] Buitrago-Bitar M.A., Enríquez-Valencia A.L., Londoño-Caicedo J.M., Muñoz-Flórez J.E., Villegas-Estrada B., Santana-Fonseca G.E. (2020). Molecular and morphological characterization of *Musa* spp. (Zingiberales: Musaceae) cultivars. Bol. Cient. Mus. Hist. Nat. Univ. Caldas.

[11] Lamare A., Rao S.R. (2015). Efficacy of RAPD, ISSR and DAMD markers in assessment of genetic variability and population structure of wild *Musa acuminata* Colla. Physiol. Mol. Biol. Plants.

[12] Zozimo R., Ratanasut K., Boonsrangsom T., Sujipuli K. (2017). Assessment of genetic diversity among Thai banana cultivars (*Musa* spp.) based on RAPD and SRAP markers. Int. J. Biol. Sci..

[13] Boonsrangsom T., Phetnin B., Ratanasut K., Sujipuli K. (2020). Assessment of genetic diversity among *Musa* cultivars based on sequence-related amplified polymorphism technique. J. Sci. Technol..

[14] IPGRI, INIBAP/CIRAD (1996). Descriptors for Banana (Musa spp.).

[15] Amom T., Tikendra L., Apana N., Goutam M., Sonia P., Koijam A.S., Potshangbam A.M., Rahaman H., Nongdam P. (2020). Efficiency of RAPD, ISSR, iPBS, SCoT and phytochemical markers in the genetic relationship study of five native and economical important bamboos of north-east India. Phytochemistry.

[16] Ray T., Dutta I., Saha P., Das S., Roy S.C. (2006). Genetic stability of three economically important micropropagated banana (*Musa* spp.) cultivars of lower Indo-Gangetic plains, as assessed by RAPD and ISSR markers. Plant Cell Tissue Organ Cult..

[17] Vanijajiva O. (2012). The application of ISSR markers in genetic variance detection among Durian (*Durio zibethinus* Murr.) cultivars in the Nonthaburi Province, Thailand. Procedia Eng..

[18] Riupassa P.A., Chikmawati T. (2015). The Molecular diversity-based ISSR of *Durio tanjungpurensis* originating from West Kalimantan, Indonesia. Makara J. Sci..

[19] Angeliena A., Ma’ruf A., Sidiq H.A., Anggraito Y.U., Habibah N.A., Huyop F.Z., Retnoningsih A. (2019). The diversity of superior Indonesian durians based on molecular markers. AIP Conf. Proc..

[20] Kimaro D., Melis R., Sibiya J., Shimelis H., Shayanowako A. (2020). Analysis of genetic diversity and population structure of Pigeonpea (*Cajanus cajan* [L.] Millsp) accessions using SSR markers. Plants.

[21] Arumugam T., Jayapriya G., Sekar T. (2019). Molecular fingerprinting of the Indian medicinal plant *Strychnos minor* Dennst. Biotechnol. Rep..

[22] Ruangsuttapha S., Eimert K., Schröder M., Silayoi B., Denduangboripant J., Kanchanapoom K. (2007). Molecular phylogeny of banana cultivars from Thailand based on HAT-RAPD markers. Genet. Resour. Crop Evol..

[23] Suvittawat K., Silayoi B., Teinseree N., Saradhuldhat P. (2014). Growth and yield of eight ‘NAM WA’ (ABB) banana in Thailand. Acta Hortic..

[24] Njuguna J., Nguthi F., Wepukhulu S., Wambugu F., Gitau D., Karuoya M., Karamura D. (2008). Introduction and evaluation of improved banana cultivars for agronomic and yield characteristics in Kenya. Afr. Crop Sci..

[25] Youssef M., James A.C., Rivera-Madrid R., Ortiz R., Escobedo-GraciaMedrano R.M. (2011). Musa genetic diversity revealed by SRAP and AFLP. Mol. Biotechnol..

[26] Igwe D.O., Ihearahu O.C., Osano A.A., Acquaah G., Ude G.N. (2021). Genetic diversity and population assessment of *Musa* L. (Musaceae) employing CDDP markers. Plant Mol. Biol. Rep..

[27] Igwe D.O., Ihearahu O.C., Osano A.A., Acquaah G., Ude G.N. (2022). Assessment of genetic diversity of *Musa* species accessions with variable genomes using ISSR and SCoT markers. Genet. Resour. Crop Evol..

[28] Borborah K., Saikia D., Rehman M., Islam M.A., Mahanta S., Chutia J., Borthakur S.K., Tanti B. (2020). Comparative analysis of genetic diversity in some non-commercial cultivars of *Musa* L. from Assam, India, using morphometric and ISSR markers. Int. J. Fruit Sci..

